# Longitudinal behavioral and neuropsychiatric changes and their MRI correlates in predementia *C9orf72* and *GRN* mutation carriers

**DOI:** 10.1177/13872877251350684

**Published:** 2025-06-19

**Authors:** Hyunwoo Lee, Atri Chatterjee, Ian RA Mackenzie, Imogene Scott, Mirza Faisal Beg, Karteek Popuri, Dana Wittenberg, Rosa Rademakers, Ging-Yuek Robin Hsiung

**Affiliations:** 1 Division of Neurology, Department of Medicine, University of British Columbia, Vancouver, BC, Canada; 2 Department of Pathology and Laboratory Medicine, University of British Columbia, Vancouver, BC, Canada; 3 School of Engineering Science, Simon Fraser University, Burnaby, BC, Canada; 4 Department of Computer Science, Memorial University of Newfoundland, St John's, NL, Canada; 5 Applied and Translational Neurogenomics, VIB Center for Molecular Neurology, VIB, Antwerp, Belgium; 6 Department of Neuroscience, Mayo Clinic, Jacksonville, FL, USA

**Keywords:** Alzheimer's disease, behavior, frontotemporal dementia, genetic, magnetic resonance imaging, neuropsychiatric symptoms, neuropsychological measures

## Abstract

**Background:**

Neuropsychiatric symptoms (NPS) progress differently among individuals with autosomal dominant familial frontotemporal dementia (FTD) caused by genetic mutations in granulin (*GRN*+) or chromosome 9 open reading frame 72 (*C9orf72*+).

**Objective:**

To determine whether these differences begin prior to the onset of dementia, we compared the longitudinal rates of change of NPS among *C9orf72*+, *GRN*+, and noncarrier controls in the predementia phase. Additionally, we assessed whether the NPS changes were correlated with gray matter (GM) volume loss or white matter signal abnormalities (WMSAs) on magnetic resonance imaging (MRI).

**Methods:**

Eighty-two participants (N = 10 *GRN*+, N = 23 *C9orf72*+, N = 49 noncarriers) were followed using various NPS rating scales for an average of 7.8 years. Group differences were compared using generalized linear mixed-effects models. GM volume and WMSA volumes were measured on 42 participants (N = 8 *GRN*+, N = 11 *C9orf72*+, N = 23 noncarriers) who had two MRI visits. These measures were correlated with the rates of NPS score changes.

**Results:**

*C9orf72*+ showed higher rates of increase in the Beck Depression Inventory (BDI) total and the Iowa Scales of Personality Change (ISPC) dysexecutive disturbance scores versus noncarriers. *GRN*+ showed higher rates of increase in the BDI total, the ISPC total, and the emotional/social disturbance scores versus noncarriers; and higher rates of increase in the ISPC emotional/social personality and distressed disturbance scores versus *C9orf72*+. Across all groups, faster WMSA accumulation correlated with higher rates of increase in the Neuropsychiatric Inventory Questionnaire total score.

**Conclusions:**

Changes in NPS differ among *C9orf72*+, *GRN*+, and noncarrier controls prior to the onset of overt FTD.

## Introduction

Frontotemporal dementia (FTD) is characterized by progressive changes in behavior and/or language, often in combination with motor symptoms.^1^ Although not a defining feature, neuropsychiatric symptoms (NPS), such as delusions, hallucinations, depression and mania, are also relatively common in FTD^2^ and may manifest early.^3^ In fact, a psychiatric diagnosis precedes dementia in approximately 51% of behavioral variant FTD (bvFTD), 24% of semantic variant primary progressive aphasia (svPPA) and 12% of nonfluent variant primary progressive aphasia (nfvPPA) patients.^4^ While most people with dementia experience NPS and the specific symptoms can overlap across conditions, some factors that may distinguish FTD include an earlier age of onset (typically 50s–60s),,^5,6^ early behavioral and personality changes (e.g., disinhibition, apathy, loss of empathy, compulsions) in bvFTD, or language deficits in svPPA and nfvPPA.^7^

A significant proportion of FTD cases are familial, with the most common genetic causes being autosomal dominant mutations in the microtubule-associated protein tau gene (*MAPT*), progranulin (*GRN*), and chromosome 9 open reading frame 72 (*C9orf72*) genes.^8^ In particular, the cases caused by *C9orf72* and *GRN* are characterized by abnormal accumulation of Transactive response DNA-binding protein 43 (TDP-43) in the cytoplasm, which represents the key pathological hallmark of frontotemporal lobar degeneration-TDP (FTLD-TDP), the most common neuropathological subtype of FTD.^8^ The C9orf72 protein is known to be involved in cellular processes including vesicle trafficking, lysosome homeostasis, mammalian target of rapamycin complex 1 (mTORC1) signaling and autophagy.^9^ *C9orf72* is on the minus strand of chromosome 9 (at 9p21.2), and has 11 exons that yield 5 mRNA splice variants through alternative splicing.^10^ Disease is associated with abnormal expansion of a hexanucleotide (GGGGCC) repeat in a non-coding region of the *C9orf72* gene. Multiple potential pathogenic mechanisms are recognized, including loss-of-function of the C9orf72 protein, the formation of toxic RNA foci and aberrant translation of the massively expanded repeat region which generates a set of five dipeptides.^10^ The penetrance of the *C9orf72* expansion is recognized as strongly age-dependent. While the median age of symptom onset is estimated at approximately 58 years, near-complete penetrance is observed by the early 80s.^11^ The progranulin protein is involved in the regulation of lysosomal biogenesis, inflammation, repair, stress response, and aging.^12^ *GRN* is encoded on the plus strand of chromosome 17 (at 17q21.31), and comprises a total of 13 exons.^13^ The most common mechanism of *GRN*-related FTD involves loss-of-function nonsense, splicing, and frameshift mutations causing the creation of mRNAs containing premature termination codons that get degraded by non-sense mediated mRNA decay, which leads to reduction of functional progranulin protein (i.e., haploinsufficiency).^13,14^ The penetrance of the *GRN* mutation is recognized as age-dependent. The mean age at onset is approximately 60 years, with an observed range of 35 to 89 years. Penetrance reaches 90% by the age of 70 years.^15,16^

FTD patients with genetic mutations show varying profiles of behavioral features and NPS, depending on the specific underlying genetic variant. For example, symptomatic carriers of mutations in the microtubule-associated protein tau gene (*MAPT*) tend to show more severe disinhibition and compulsive behavior; whereas, those with mutations in the chromosome 9 open reading frame 72 (*C9orf72*) or granulin gene (*GRN*) have increased frequencies of delusions and hallucinations.^17–19^ Magnetic resonance imaging (MRI) findings suggest that the progression of NPS in patients with FTD and other dementia subtypes may be associated with structural changes, such as gray matter (GM) atrophy^20,21^ or white matter signal abnormalities (WMSA).^22^–^24^

In this study, we investigated a longitudinal cohort of genetic FTD to better understand the progression of NPS and their anatomical correlates in the pre-dementia stage of disease. We hypothesized that (1) *GRN* and *C9orf72* mutation carriers would have an increased burden and rate of progression of NPS prior to the onset of dementia compared to their family members who do not carry the respective mutation (non-carriers), (2) that pre-dementia NPS measures would be associated with specific patterns of GM volume loss and/or WMSA on structural MRI, and (3) that there would be distinct NPS profiles and corresponding imaging changes between the two mutation carrier groups.

## Methods

### Participants

Participants were from the University of British Columbia Familial FTD study, which is an ongoing single-center longitudinal observational study of a Canadian cohort of familial FTD associated with autopsy-proven transactive response DNA binding protein 43 kDa (TDP-43) pathology (FTLD-TDP). The study was approved by the University of British Columbia Clinical Ethics Review Board. All participants provided written informed consent. Study recruitment began in January 2006, and the inclusion/exclusion criteria were described previously.^25^ For the present study, we compared (1) carriers of pathogenic mutations in *GRN* (*GRN+*), (2) carriers of abnormal *C9orf72* repeat expansions (*C9orf72+*), and (3) their family members who did not carry the respective mutations (non-carriers, *GRN*- and *C9orf72*- combined). All genetic analyses were conducted in the lab of Dr Rosa Rademakers at The Mayo Clinic, Jacksonville, Florida.

Six pathogenic *GRN* mutations were identified in the participating families included in this study: c.1252C > T, c.1157G > A, c.90_91insCTGC, c.1428_1431delGGAT, c.933 + 1G > A, and c.463-1G > T. Among the *GRN* carriers, one had the c.1252C > T mutation, eight had c.90_91insCTGC, and one had c.933 + 1G > A. Furthermore, 21 participants were noncarriers but had family members known to carry one of the six identified mutations. Nonsense variants included: c.1252C > T, a C > T substitution at nucleotide position 1252 (codon 418) within exon 11, predicting the protein change p.Arg418Ter, and c.1157G > A, a G > A substitution at nucleotide position 1157 (codon 386) within exon 10, predicting the protein consequence p.W386X; both create premature stop codons.^13,14,26^ Frameshift mutations included: c.90_91insCTGC, an insertion predicted to result in p.C31LfsX34 with a premature stop after 34 residues, and c.1428_1431delGGAT, a four base-pair deletion located within exon 12 that causes a frameshift beginning at codon 476, leading to a predicted p.E476DfsX14 protein change and a premature stop codon.^13,26,27^ Splice site mutations predicted to disrupt normal mRNA splicing included: c.933 + 1G > A, occurring at nucleotide position +1 within intron 9 (immediately following coding nucleotide 933), and c.463-1G > T, a substitution located at the canonical splice acceptor site of intron 5 (position −1 relative to exon 6).^13,14,26^ Despite this variety in mutation type and location, all these identified pathogenic *GRN* mutations ultimately lead to the same predicted biological outcome: a loss-of-function of the affected *GRN* allele, resulting in progranulin haploinsufficiency. This aligns with the established haploinsufficiency mechanism for *GRN*-related FTD. Although predicting the exact age of FTD onset is challenging due to the small number of the participants, most carriers are expected to develop symptoms later in life due to the high penetrance rates.^15,16^

*C9orf72* gene expansions exceeding 30 repeats are generally considered pathogenic and associated with FTD and/or amyotrophic lateral sclerosis.^28^ However, accurately measuring these expansions is technically challenging due to both intra-individual (cross-tissue) and inter-individual variation in repeat numbers.^29–31^ Furthermore, a definitive link between the specific repeat expansion size and resulting clinical or psychiatric phenotypes has not yet been established.^29,31^ In our study, all *C9orf72*-positive participants had repeat sizes well within the pathogenic range, exceeding 100 repeats.

All participants underwent baseline and annual follow-up neurological, neuropsychological and neuroimaging examinations. Data from the initial visit in 2006 through the 2022 assessments were used for this analysis. After each assessment, participants were classified as asymptomatic, clinically symptomatic but not demented (CSND), or demented (FTD), by a consensus meeting involving the study neurologist and neuropsychologist.^25^

The analysis of NPS included a total of 82 participants with a baseline diagnosis of asymptomatic or CSND (N = 10 *GRN*+ from three families, N = 23 *C9orf72*+ from seventeen families, N = 49 non-carriers from twenty families; mean follow-up of 7.8 ± 5.1 years) (Table 1). Fifteen families contributed to both carriers and non-carriers groups, three for *GRN*+ and twelve for *C9orf72*+ . In 12 of these 15 families, mutation carriers had immediate family members (e.g., siblings) from the same generation assigned in the non-carrier comparator group. Two *GRN*+ mutation carriers developed symptomatic FTD during the follow-up; however, only observations collected during their pre-dementia stage was included in the analysis. A subset of 42 participants with MRI scans acquired during their first two visits were available for the analysis of NPS-MRI correlations (N = 8 *GRN+* from two families, N = 11 *C9orf72+* from ten families, N = 23 non-carriers from eight families; mean follow-up of 9.1 ± 3.8 years) (Table 2).

**Table 1.** Baseline demographic characteristics and NPS measures.

	*GRN+* (N = 10)	*C9orf72+* (N = 23)	Non-carriers (N = 49)
Age in years, mean ± SD, [range]	50.5 ± 8.7 [36–64]	47.1 ± 10.8 [22–74]	53.3 ± 11.3 [22–81]
Female (%)	80	57	53
Education in years, mean ± SD	12.5 ± 2.3	14.1 ± 2.9	13.8 ± 2.9
3MS Score, mean ± SD	98.1 ± 2.1	98.0 ± 2.8	97.6 ± 2.5
NPI-Q Score, mean ± SD	6.8 ± 8.0	4.2 ± 6.7	4.5 ± 8.8
FBI Score, mean ± SD	0.6 ± 1.0	0.9 ± 1.6	0.4 ± 1.0
ISPC Residualized, mean ± SD	0.2 ± 0.9	−0.2 ± 1.0	−0.2 ± 1.1
BDI Score, mean ± SD (N = 71)	11.0 ± 9.7	8.3 ± 8.8	5.4 ± 4.8
Follow-up Duration, mean ± SD	10.3 ± 5.0	7.2 ± 4.5	7.6 ± 5.4

Groups were not significantly different after post-hoc comparisons.

BDI: Beck Depression Inventory; FBI: Frontal Behavioral Inventory; ISPC: Iowa Scales of Personality Change; NPI-Q: Neuropsychiatric Inventory Questionnaire; NPS: Neuropsychiatric symptoms.

**Table 2.** Baseline demographic characteristics and NPS measures of participants with longitudinal MRI.

	*GRN+* (N = 8)	*C9orf72+* (N = 11)	Non-carriers (N = 23)
Age in years, mean ± SD, [range]	50.1 ± 8.9 [36–64]	46.0 ± 9.1 [22–58]	52.1 ± 7.6 [33–66]
Female (%)	87.5	45.5	52.2
Education in years, mean ± SD	12.1 ± 1.3	14.6 ± 3.1	13.6 ± 3.0
3MS Score, mean ± SD	98.1 ± 2.0	98.4 ± 1.8	98.0 ± 2.2
NPI-Q Score, mean ± SD	7.3 ± 8.1	4.8 ± 7.4	3.3 ± 7.7
FBI Score, mean ± SD	0.6 ± 0.9	1.2 ± 1.6	0.4 ± 1.1
ISPC Residualized, mean ± SD	0.3 ± 0.8	−0.1 ± 1.0	−0.2 ± 1.1
BDI Score, mean ± SD	11.4 ± 9.8	10.5 ± 9.7	6.0 ± 5.2
Baseline volume of cortical gray matter, normalized using total intracranial volume, mean ± SD	30.5 ± 4.7^a^	26.6 ± 2.4	25.8 ± 3.2
Baseline volume of white matter signal abnormalities, normalized using total intracranial volume, mean ± SD	0.1 ± 0.03	0.1 ± 0.04	0.1 ± 0.05
Follow-up Duration, mean ± SD	12.1 ± 3.5	9.7 ± 4.9	10.1 ± 4.8

^a^ *GRN* carriers versus Non-carriers p < 0.05, after post-hoc comparisons.

BDI: Beck Depression Inventory; FBI: Frontal Behavioral Inventory; ISPC: Iowa Scales of Personality Change; MRI: magnetic resonance imaging; NPI-Q: Neuropsychiatric Inventory Questionnaire; NPS: Neuropsychiatric symptoms.

### Assessment of neuropsychiatric symptoms

At each annual visit, NPS were assessed using rating scales that included: the Neuropsychiatric Inventory Questionnaire (NPI-Q),^32,33^ the Iowa Scales of Personality Change (ISPC),^34^ the Beck Depression Inventory (BDI),^35^ and the Frontal Behavioral Inventory (FBI).^36^

The NPI-Q is an informant-based interview that encompasses 12 domains, where for each item, the informant is asked whether the symptoms were absent or present in the last month and to rate the severity from 1–3. The ISPC is an informant-rated scale that provides standardized assessment of 30 characteristics concerning disturbances in mood, affect, drive, social/interpersonal behavior, adaptive functioning, and cognitive functions. Ratings for each characteristic are made at two different time points: “Now” that rates a subject's functioning over the past year, and “Before” that rates their performance five years prior to the respective visit. Each characteristic is scored on a 7-point scale (1–7). As suggested by Barrash and colleagues,^37^ we have calculated the residualized disturbance scores by regressing the “Before” ratings against the “Now” ratings: this yields the z-scores for the current level of personality disturbance, with the variance due to past performances covaried out statistically.^37^ The BDI is a self-reported, 21-item inventory that assesses characteristic attitudes and symptoms of depression over 21 items, which are each scored from 0–3. The FBI is an informant-rated scale with the primary purpose of differentiating bvFTD from other types of dementias. The scale encompasses 12 negative and 12 positive behaviors, each scored from 0–3.

Typically for these rating scales, the severity score is noted for each item and then summed over all domains to calculate the total severity score that reflects the overall level of symptoms. Additionally, for the NPI-Q and ISPC, previous factor analyses have identified clinically relevant sub-syndromes/ sub-dimensions derived from related domains/items. For example, the NPI-Q can include hyperactivity, affective, psychosis, and apathy subsyndromes,^38^ and the ISPC can include emotional/social, dysexecutive, hypoemotional, and distressed personality disturbances.^37^ In the current analysis, we have also included the severity scores for the NPI-Q subsyndromes and the ISPC personality subdimensions, as outlined in Table 3. For each subsyndrome or subdimension, the associated individual item severity scores were summed to calculate the severity scores.

**Table 3.** Neuropsychiatric Inventory Questionnaire (NPI-Q) subsyndromes and the Iowa Scales of Personality Change (ISPC) personality subdimensions included in the current analysis.

Adapted from Aalten et al., Dement Geriatr Cogn Disord 2007	NPI-Q Domain	NPI-Q Subsyndromes
	**Agitation**	**Hyperactivity**
	**Disinhibition**	
	**Irritability**	
	**Aberrant Motor Behavior**	
	**Anxiety**	**Affective**
	**Depression**	
	**Delusions**	**Psychosis**
	**Hallucinations**	
	**Nighttime Behavior**	
	**Apathy**	**Apathy**
	**Appetite**	
Adapted from Barrash et al., Cortex 2022	ISPC Item	Personality Dimension
	**Irritability**	**Emotional/Social **
	**Impatience**	
	**Socially Inappropriate Behavior**	
	**Insensitivity**	
	**Inflexibility**	
	**Lack of Planning**	**Dysexecutive **
	**Lack of Persistence**	
	**Perseverative Behavior**	
	**Lack of Initiative**	
	**Blunted Affect**	**Hypoemotional **
	**Apathy**	
	**Social Withdrawal**	
	**Anxiety**	**Distressed **
	**Depression**	
	**Easily Overwhelmed**	

Individual item severity scores were summed to calculate the subsyndrome or subdimension severity scores.

### MRI acquisition

MRI data were acquired on a 1.5T GE Signa scanner at the UBC Hospital MRI Research Centre, using the following imaging parameters: (1) Localizers (0:25 min): sagittal/coronal and axial; Fast Gradient TR 5.4, TE 1.6, 1 average, Field of View (FOV) 22 cm, 256 × 128 matrix; 2) 3D T1-Fast Spoiled Gradient Echo IR Prepped (8:35 min): TR/TE (ms) = 10.3/4.8, 8° flip angle, 166 × 256 × 256, 1.0 × 0.98 × 0.98 mm^3^, FOV 166; 3) Proton density (PD)/T2 Dual (4:00 min), axial, TR = 2800, TE = 30/90, 90° flip angle, 256 × 256 × 35, 0.86*0.86*5.0 mm^3^, FOV 220; 4) Diffusion Tensor Imaging (11:42 min): axial, spin-echo echo-planar imaging, TR =  13,000, TE = min, 25 directions, 2 averages, 256 × 256 × 48, 1.25 × 1.25 × 2.50 mm^3^, FOV 320.

For the NPS-MRI correlations, changes in the MRI scans acquired at the time of the first and second clinical visits were analyzed (mean interval of 2.4 ± 0.7 years).

### MRI processing

All T1-weighted images were visually checked for quality and then processed using the FreeSurfer 5.3 pipeline, which provides cortical reconstruction and volumetric segmentation.^39,40^ All FreeSurfer outputs were manually examined and corrected for errors, as per FreeSurfer's quality-control guidelines. Total intracranial volumes (TIV) were calculated with a multi-atlas label fusion method.^41^

Bilateral frontal, temporal, parietal and occipital lobe GM volumes were calculated by combining the corresponding Desikan-Killiany region-of-interest (ROI) labels using FreeSurfer's ‘mri_annotation2label’ and ‘mri_mergelabels’ functions.^42^ This resulted in four cortical lobar GM volumes per time point, per participant.

As an indicator of WMSA, we used hypointensities on T1-weighted MRI (T1-HypoWMSA) that were segmented as part of the FreeSurfer volumetric outputs. While we recognize that WMSAs are conventionally represented by hyperintensities on T2-weighted MRI, it has been shown that the volumes of FreeSurfer-derived WM hypointensities are strongly correlated with the volumes of hyperintensities on Fluid-Attenuated Inversion Recovery images.^43^ Similar to the lobar definitions of cortical GM volumes, we defined frontal, temporal, parietal and occipital lobe WM using FreeSurfer's ‘mri_annotation2label’ and ‘mri_aparc2aseg’ functions. Then, the lobar-specific T1-HypoWMSA volumes were calculated by only accounting for those placed within the respective lobes. This resulted in four lobar WMSA volumes per time point, per participant.

For each participant, annualized percentage changes between the two MRI visits were calculated for each of the lobar-specific cortical GM and T1-HypoWMSA volumes.

### Statistical analysis

All statistical analyses were conducted using SAS v9.4 and R v3.6.2. The significance level was set at p < 0.05 (two-tailed) for all comparisons. The Shapiro-Wilk test was used to examine whether continuous variables were normally distributed.

### Baseline demographic and NPS characteristics (NPS and NPS-MRI analyses)

The Chi-square test was used to evaluate Bonferroni-corrected group differences in the sex ratio. A one-way Analysis of Variance (ANOVA) was used to assess baseline differences in age, education and Modified Mini-Mental State (3MS) scores among *C9orf72+*, *GRN+* and noncarriers. Baseline NPI-Q, FBI and BDI total scores were compared among *C9orf72+*, *GRN+* and noncarriers using the Kruskal–Wallis test as the scores were not normally distributed. ISPC residualized scores were compared using a one-way ANOVA. Pairwise post-hoc comparisons were conducted using Dunnett's method with noncarriers as the reference.

### Longitudinal rates of change in NPS measures (NPS analysis)

We conducted a genetic group-wise comparison of the longitudinal rates of change in each NPS rating scale using generalized linear mixed-effects (GLME) models with subject-specific random intercepts and slopes to account for unobserved characteristics that are shared within a participant but may vary between participants. In the event of unsuccessful model convergence, the random slopes were removed and the model included only random intercepts. Independent variables included age, sex, baseline assessment scores of the respective NPS ratings scale, years from baseline, genetic status and an interaction term between genetic status and years from baseline. Years from baseline was used as the time variable. A second-order polynomial quadratic term was included to model the potential non-linearity of the change rates, and the significance of this term was used to determine the group differences in the rates of NPS scale change. Accordingly, group difference was determined based on the statistical significance of the quadratic interaction term. The NPI-Q, FBI and BDI models assumed over-dispersed Poisson distribution due to the frequent zero-valued scores in the respective rating scale; the ISPC models assumed normal distribution of the residualized scores.

### MRI-based volumetric characteristics (NPS-MRI analysis)

Baseline total volumes of cortical GM and T1-HypoWMSA among *C9orf72+*, *GRN* *+* and non-carriers were compared using a general linear model (GLM) adjusted for age and sex. Annualized percentage changes in the MRI volumetric measures were compared among *C9orf72+*, *GRN+* and non-carriers using a similar GLM, with additional adjustment for TIV-normalized baseline volumes of the T1-HypoWMSA (for WMSA comparison) or cortical GM (for GM comparison). Dunnett's method was used for pairwise post-hoc comparisons with non-carriers as the reference.

### Association between NPS change rates and MRI measures (NPS-MRI analysis)

We adopted a two-stage approach. First, we calculated subject-specific rates of change of the NPS measures using linear mixed-effects models with the time variable and random intercepts and slopes. Second, for each NPS measure, we used two GLMs to evaluate the relationship between the lobar-specific MRI measure changes over the first two visits (dependent variables) and the change rates of the total severity scores over all visits (independent variable) The first model included lobar GM volume changes and the second model included lobar T1-HypoWMSA volume changes. Both models were adjusted for age, sex, baseline scores of the respective NPS ratings scale, and TIV-normalized baseline volumes of the total WM and T1-HypoWMSA (for WMSA model) or cortical GM (for the GM model).

Baseline assessment scores (of the respective NPS ratings scale) were included as they were expected to influence the longitudinal rates of change. Potential multi-collinearity of the dependent variables was assessed in terms of variance inflation factors. For all models, we first conducted an omnibus F-test to examine whether there were any significant differences in the means among the genetic groups. If the F-test was significant, we assessed an effect test of the MRI volume change variables. If the F-test was not significant, we did not proceed with the effect test.

## Results

### Baseline demographic and NPS characteristics (NPS and NPS-MRI analyses)

For both analyses, *C9orf72+*, *GRN+* and non-carriers were not significantly different in terms of demographic variables or baseline NPS measures (Tables 1 and 2).

### Longitudinal mixed effects model of NPS change rates (NPS analysis)

**Figure 1.**
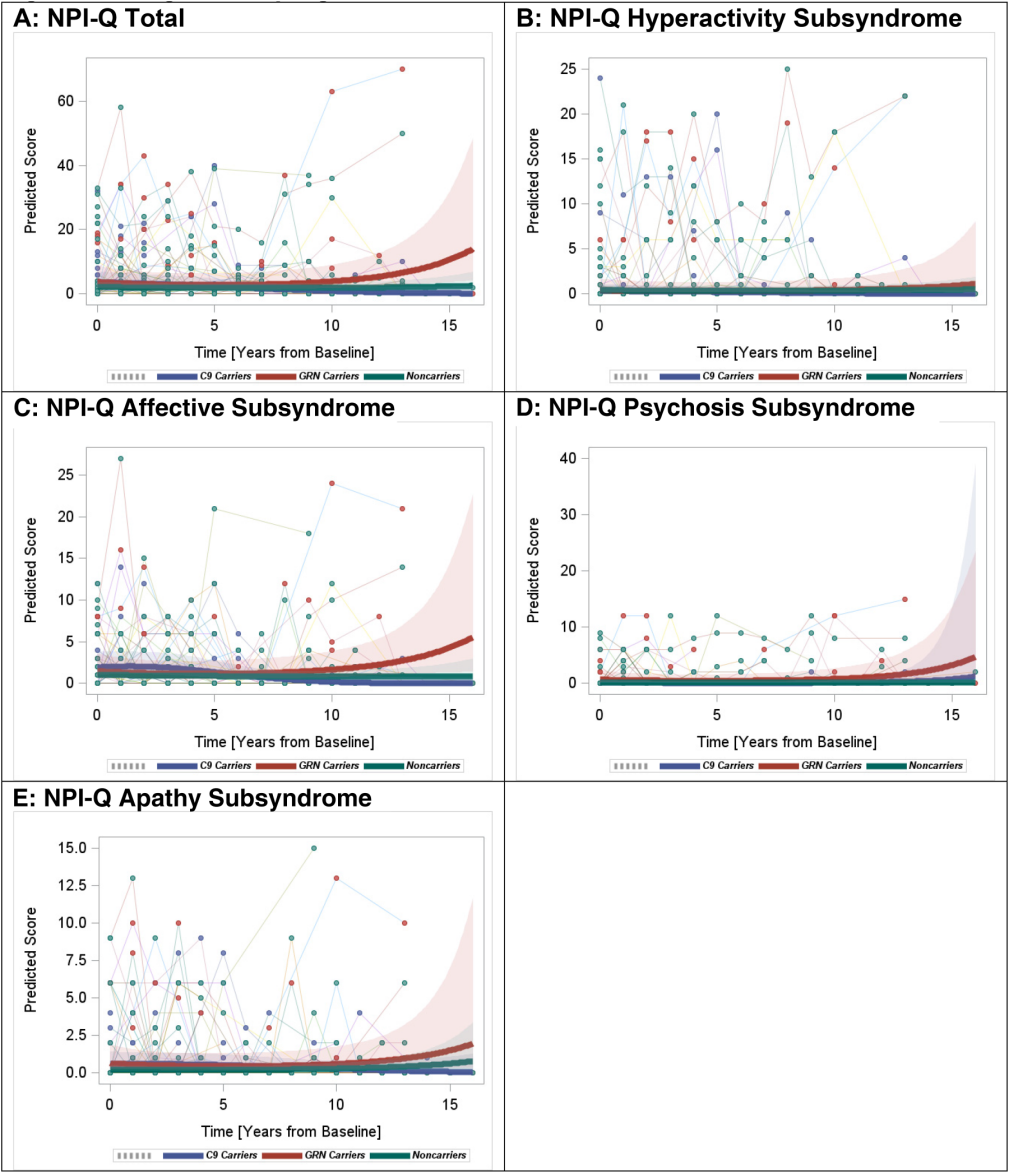
Longitudinal progression of the NPI-Q scores.

*NPI-Q: Total Scores*: Longitudinal rates of change in the NPI-Q total scores were not significantly different among *C9orf72+*, *GRN+* and non-carriers, although *GRN+* showed a trend towards an overall increase in the total score (p = 0.06) (Figure 1A).

Generalized mixed linear effects models. Thick lines indicate group-specific models derived from the estimates of fixed effects. Shaded colors indicate 95% confidence intervals. Thin lines show the actual data for individual participants. NPI-Q: Neuropsychiatric Inventory Questionnaire.

#### Subsyndromes

Examination of the NPI-Q subsyndromes showed that the longitudinal rates of hyperactivity, psychosis, affective, and apathy symptom changes were not significantly different between mutation carriers and non-carriers. However, *GRN+* showed higher rates of increase in the affective symptoms compared to *C9orf72+* (p = 0.03) (Figure 1B-E). *GRN+* showed an overall trend towards increases in all four subsyndromes, which explained the overall trend of increase in the NPI-Q total score.

#### ISPC: total scores

**Figure 2.**
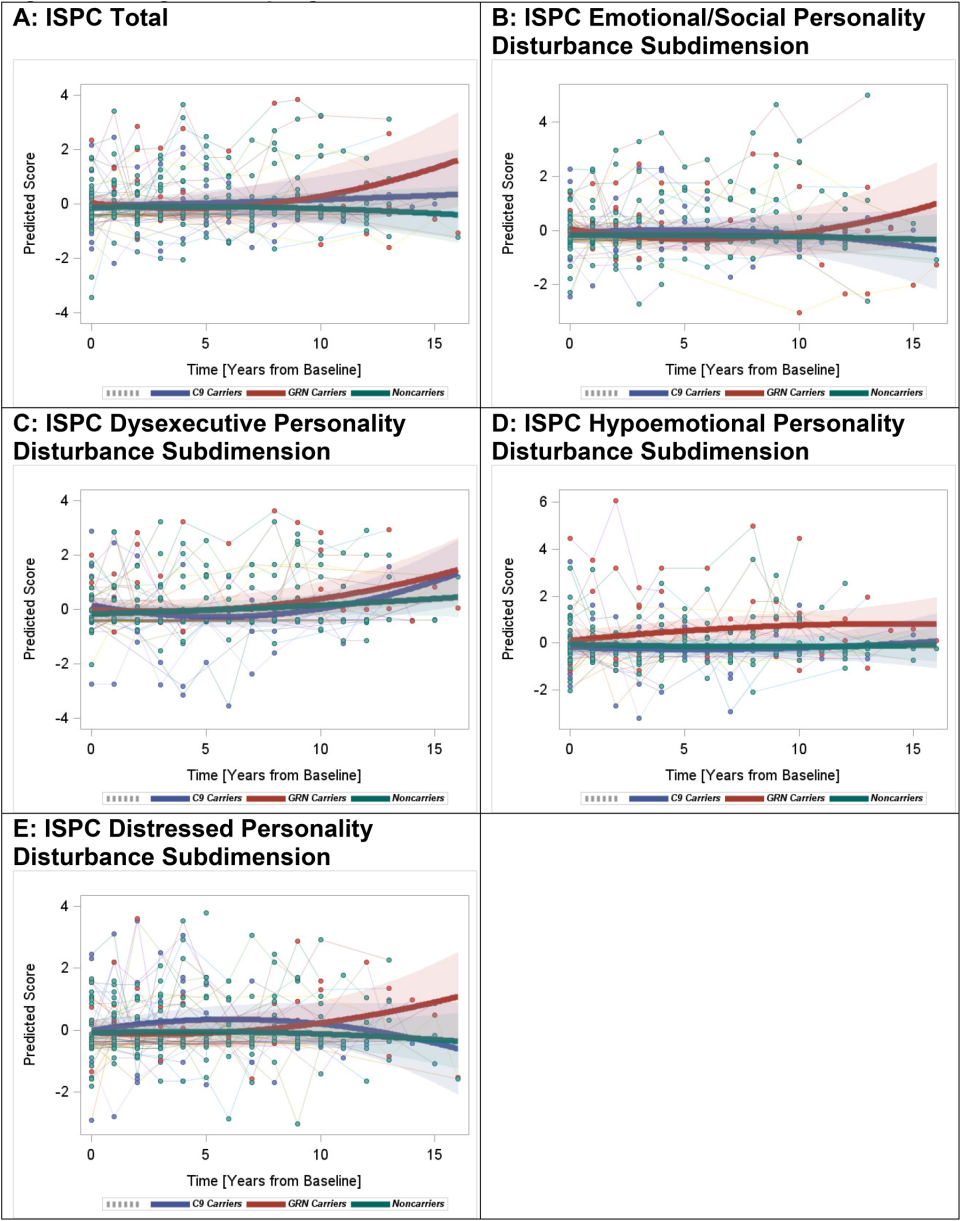
Longitudinal progression of the ISPC scores.

Rates of change in the ISPC total score were significantly different among the groups, mainly driven by significant increases observed in *GRN+* versus non-carriers (quadratic interaction β=0.01, p = 0.003) as well as versus *C9orf72+* (quadratic interaction β = 0.01, p = 0.04) (Figure 2A). The changes observed in *C9orf72+* were similar to those in non-carriers.

Generalized mixed linear effects models. Thick lines indicate group-specific models derived from the estimates of fixed effects. Shaded colors indicate 95% confidence intervals. Thin lines show the actual data for individual participants. ISPC: Iowa Scales of Personality Change.

#### Subdimensions

Comparison of the mutation carriers versus non-carriers revealed significant group effects in the emotional/social personality and the dysexecutive personality disturbances. Specifically, increased rates of change in the emotional/social personality disturbance were observed in *GRN+* versus non-carriers (quadratic interaction β = 0.01, p = 0.008) but not in *C9orf72+* versus non-carriers (Figure 2B). In contrast, rates of increase in the dysexecutive personality disturbance were higher in *C9orf72+* versus non-carriers (quadratic interaction β = 0.01, p = 0.01) (Figure 2C). *GRN+* also showed a trend towards increases in dysexecutive personality disturbance; however, this was not statistically significant. Rates of change in the hypoemotional personality and the distressed personality disturbances were not significantly different between mutation carriers and non-carriers (Figure 2D and E).

Comparing the two mutation carrier groups, *GRN+* showed higher rates of increase in the emotional/social personality (quadratic interaction β = 0.02, p = 0.002) and the distressed personality (quadratic interaction β = 0.02, p = 0.03) disturbances versus *C9orf72+* . Changes in the dysexecutive and the hypoemotional personality disturbances were not significantly different between the two carrier groups.

#### BDI

**Figure 3.**
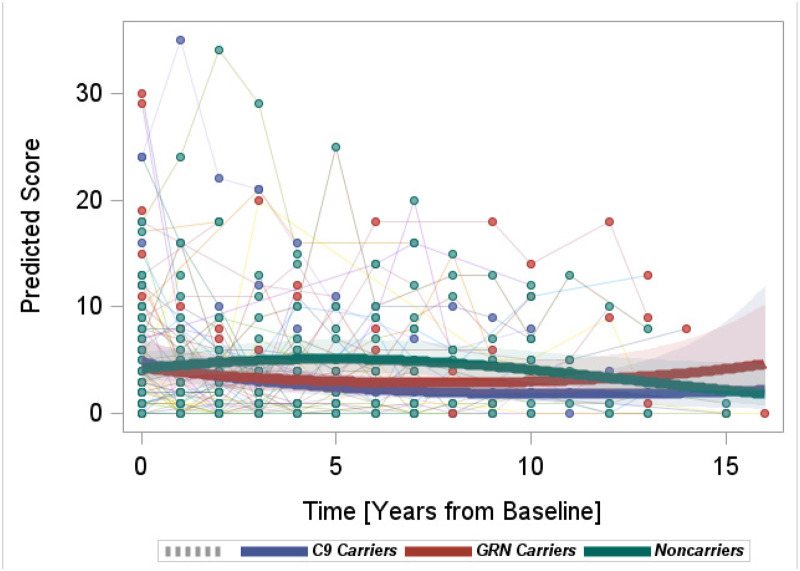
Longitudinal progression of the BDI scores.

Non-carriers showed an overall decrease in BDI scores, while both mutation carrier groups showed significantly increasing rates of BDI score changes compared to non-carriers (*C9orf72+* versus non-carriers, quadratic interaction β = 0.02, p = 0.03; *GRN* *+* versus non-carriers, quadratic interaction β = 0.02, p = 0.006) (Figure 3). While the average scores towards the end of the model were comparable to those at the beginning of the model, the direction in the change rates implied future increases in the scores in *GRN+* and *C9orf72+* .

Generalized mixed linear effects models. Thick lines indicate group-specific models derived from the estimates of fixed effects. Shaded colors indicate 95% confidence intervals. Thin lines show the actual data for individual participants. BDI: Beck Depression Inventory.

**Figure 4.**
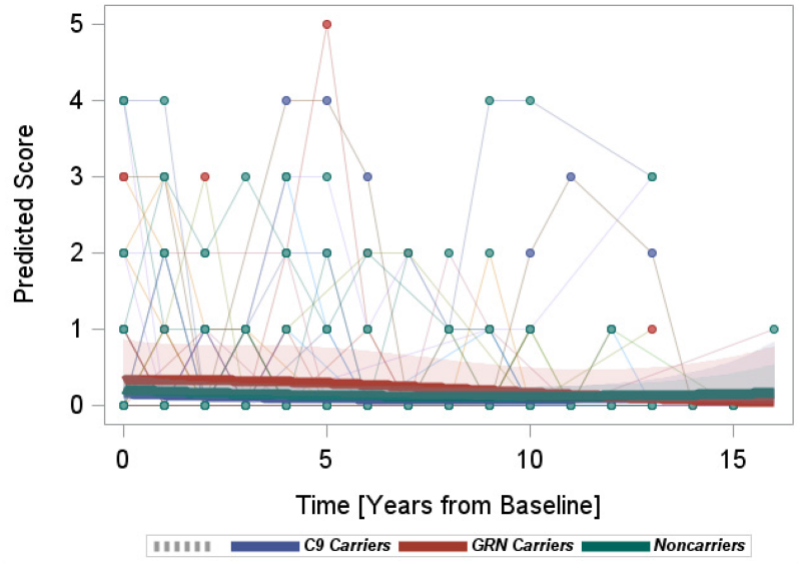
Longitudinal progression of the FBI scores.

*FBI:* Overall, the model suggested minimal changes in FBI scores for all three groups. Also, there were no significant differences in the rates of FBI change among the groups (Figure 4).

Generalized mixed linear effects models. Thick lines indicate group-specific models derived from the estimates of fixed effects. Shaded colors indicate 95% confidence intervals. Thin lines show the actual data for individual participants. FBI: Frontal Behavioral Inventory.

### MRI-based volumetric characteristics (NPS-MRI analysis)

Pairwise comparisons showed that *GRN+* had higher baseline volumes of cortical GM compared to non-carriers (p = 0.03; adjusted for age and sex); whereas *C9orf72+* and non-carriers were not significantly different (Table 2). However, there were no significant group differences in the annualized percentage changes in the cortical GM volumes over the two MRI time points (Table 4). All groups had similar baseline total volumes of T1-HypoWMSA (Table 2). As reported previously,^44^ increases in the total T1-HypoWMSA volumes were significantly higher in *GRN*+ (*GRN+* =15.4%/year, *C9orf72+* = 2.7%/year, non-carriers = 2.8%/year; *GRN+* versus non-carriers p = 0.003, *C9orf72+* versus non-carriers p > 0.9 after Dunnett's post-hoc test; adjusted for age, sex, and baseline volume); an effect mainly driven by the changes in the frontal region (Table 4).

**Table 4.** Change in MRI measures over the first two annual visits.

	Region	Mean ± SE^a^
Cortical Gray Matter	Frontal	*GRN+*: −1.1 ± 0.8 *C9orf72+*: −1.2 ± 0.6 Non-carriers: −0.9 ± 0.4
	Temporal	*GRN+*: −0.2 ± 0.8 *C9orf72+*: −0.3 ± 0.6 Non-carriers: −0.1 ± 0.4
	Parietal	*GRN+*: −0.7 ± 0.7 *C9orf72+*: −1.4 ± 0.6 Non-carriers: −0.5 ± 0.4
	Occipital	*GRN+*: −0.3 ± 0.7 *C9orf72+*: −1.1 ± 0.6 Non-carriers: −0.7 ± 0.4
	Whole Brain (across all lobes)	*GRN+*: −0.7 ± 0.6 *C9orf72+*: −1.0 ± 0.5 Non-carriers: −0.6 ± 0.4
White Matter Signal Abnormalities (Hypointensities on T1-w MRI)	Region	Mean ± SE^a^
	Frontal	*GRN+*: 16.8 ± 3.8^b^ *C9orf72+*: 2.4 ± 3.2 Non-carriers: 1.9 ± 2.2
	Temporal	*GRN+*: −2.6 ± 10.6 *C9orf72+*: 8.5 ± 9.0 Non-carriers: 4.8 ± 6.0
	Parietal	*GRN+*: 13.1 ± 5.5 *C9orf72+*: 7.2 ± 4.6 Non-carriers: 5.1 ± 3.1
	Occipital	*GRN+*: 29.6 ± 11.7 *C9orf72+*: 5.0 ± 9.9 Non-carriers: 17.0 ± 6.6
	Whole Brain (across all lobes)	*GRN+*: 15.3 ± 3.1^b^ *C9orf72+*: 3.7 ± 2.7 Non-carriers: 2.8 ± 1.8

^a^ Percentage change per year, adjusted for age, sex, and baseline volume.

^b^ *GRN* carriers versus Non-carriers or *C9orf72* carriers, p < 0.05, after post-hoc comparisons.

MRI: magnetic resonance imaging.

### Association between NPS change rates and MRI measures (NPS-MRI analysis)

We found a significant positive association between total NPI-Q score change rates and T1-HypoWMSA volume changes in the frontal lobe (β = 0.03, p = 0.04), suggesting that the increases in frontal WMSA that occurred between the first two visits predicted the NPI-Q increases over the follow-up period. Similar associations were not significant with T1-HypoWMSAs in other lobes. The model involving NPI-Q change rates and GM volume changes did not pass the omnibus F-test, likely due to the nonsignificant changes in the GM volume observed across all groups. Similarly, the omnibus F-tests were not significant for the models involving FBI, BDI and ISPC change rates and the two MRI measures. Accordingly, we did not proceed with further comparisons.

## Discussion

Utilizing a single-center cohort of *GRN* and *C9orf72* mutation carriers and their non-carrier family members, we compared the predementia rates of change of various NPS rating scales as well as their MRI correlates. Estimates from GLME models suggested there were (1) nonsignificant group differences in the rates of NPI-Q score changes among carriers and non-carriers, (2) higher rates of ISPC total and the emotional/social personality disturbance score increases in *GRN+* versus non-carriers, (3) higher rates of ISPC dysexecutive personality disturbance score increases in *C9orf72+* versus non-carriers, (4) higher rates of ISPC emotional/social personality and distressed personality disturbance score increases in *GRN+* versus *C9orf72+*, (5) higher rates of BDI total score increases in both *C9orf72+* and *GRN+* versus non-carriers, and (6) nonsignificant group differences in the rates of FBI score changes. Although there were nonsignificant differences among carriers and non-carriers in the rates of NPI-Q total score change, we found a significant association between the increases in the NPI-Q total scores and the frontal lobar T1-HypoWMSA lesion volumes.

Up to 30% of FTD patients develop NPS.^21^ The emergence of NPS can be observed early in the disease course, for example, with patients in the early stage of bvFTD developing symptoms such as apathy and disinhibition.^45^ Patients with sporadic FTD have a higher prevalence of NPS, particularly irritability and depression, compared to FTD mutation carriers.^46^

*C9orf72+* and *GRN+* each constitute approximately 5 to 10 percent of the FTD patient population. They demonstrate pathological TDP-43 inclusions and are associated with heterogeneous phenotypic presentations.^47^ Indeed, evidence shows that *C9orf72+* and *GRN+* exhibit high rates of NPS such as psychosis during their symptomatic stages.^48–52^ Longitudinally, NPS may progress in a genetic group-specific manner as exemplified by a predominance of depression and anxiety throughout the disease stages in *GRN*+ . On the other hand, *C9orf72*+ demonstrate an early predominance of anxiety followed by a late surge of auditory/visual hallucinations.^17^ We examined whether these changes can be observed in the predementia stages.

In our study, baseline NPI-Q, FBI, BDI and ISPC scores were not significantly different among predementia mutation carriers and non-carriers. On average, baseline NPI-Q, BDI, and FBI scores fell within the normal ranges established in earlier literature.^35,53,54^ A normative score for the ISPC is not well-established, although an analysis of healthy elderly individuals suggested an approximate total score of 94 or below.^55^ The closeness of the average non-residualized ISPC scores of our participants to this value may be attributable to the female preponderance among *GRN+*, as well as the relatively wide age range at baseline. This means some participants may have been closer to the expected age of onset than others. Further investigation is warranted to establish an appropriate normative range for this population, as well as to explore whether the family members in families that carry mutations may also be affected by some baseline personality abnormalities.

We utilized GLME models to compare the longitudinal rates of change in NPI-Q, ISPC, BDI, and FBI scores among mutation carriers and non-carriers. Both the NPI-Q and the FBI are widely utilized to assess NPS in FTD-related studies, although several of their items (e.g., psychotic symptoms and motor disturbances in the NPI-Q; language and semantic deficits in the FBI) are also suited to detect symptoms related to the onset of dementia. In that sense, the NPI-Q or FBI may provide less power to distinguish among predementia research participants who, by definition, would be expected to manifest mild FTD-related symptoms during the predementia stage. For instance, psychotic symptoms (e.g., hallucinations and delusions) were shown to be a factor that distinguishes FTD mutation carriers from controls,^52^ but these symptoms are unlikely to be present in the predementia phase of FTD. This may explain why the rates of NPI-Q and FBI score changes were not significantly different among predementia mutation carriers and non-carriers, and may suggest that an alternative measure may be necessary to detect group differences in the predementia FTD population.

The ISPC primarily investigates behavior and personality symptoms, including disturbed social behavior, executive/decision-making deficits, diminished motivation/hypo-emotionality, irascibility and distress^34^; all of which may also be symptoms of FTD.^34^ The first three of these factors have been shown to be sensitive to damage in the ventromedial prefrontal cortex (vmPFC),^34^ a region prominently affected in the early stages of FTD.^56^ Our findings indicate an increase in overall personality disturbances (i.e., ISPC total score) in *GRN+* . In particular, *GRN+* showed higher rates of increase versus non-carriers in the emotional dysregulation/disturbed social behavior subdimension, which encompasses impatience, irritability, inflexibility, insensitivity, and social inappropriateness. On the other hand, *C9orf72+* showed higher rates of increase versus non-carriers in the dysexecutive subdimension, which includes lack of planning, lack of persistence, perseveration, and lack of initiative. Although not significant, *GRN+* also showed a similar trend towards an overall increase in the dysexecutive subdimension as shown in Figure 2C. Executive function is a significant contributor to cognitive impairment in both mutation groups, with a stronger effect expected in symptomatic *C9orf72+* *.*^57^ Furthermore, *GRN*+ was distinguishable from *C9orf72*+ in terms of higher rates of increases in the emotional/social and the distressed personality disturbances. These longitudinal findings suggest that impaired ability to regulate emotion, as well as changes in social behavior could be among the domains of NPS that may distinguish *C9orf72*+ and *GRN*+ in the early stages of FTD. These observed differences between *GRN*+ and *C9orf72*+ carriers likely stem from different underlying mechanisms. *GRN* mutations lead to haploinsufficiency, reducing progranulin protein levels.^13,14^ As progranulin plays crucial roles in neuronal survival, lysosomal function, and neuroinflammation, reduced progranulin levels disrupt lysosomal function, leading to impaired protein degradation, accumulation of TDP-43, and exacerbation of neuroinflammation by modulating microglial activity.^13,14^ The observed increases in emotional/social and distressed personality disturbances in *GRN* + carriers might reflect the impact of progranulin deficiency on frontolimbic circuits, particularly the vmPFC and amygdala, which are critical for emotional regulation and social behavior. The greater increase in dysexecutive symptoms in *C9orf72*+ carriers might be related to the combined effects of C9orf72 protein reduction and RNA toxicity, potentially impacting dorsolateral prefrontal cortex function, a key region for executive control.^10^ Furthermore, while both *GRN* and *C9orf72* mutations lead to TDP-43 pathology, the distribution and type of TDP-43 inclusions may differ.^8^

Both *C9orf72+* and *GRN+* demonstrated a higher rate of increase in the BDI, which is in accordance with the early predominance of depression in symptomatic mutation carriers. Depression is commonly observed across the FTD subtypes with reported frequency of around 30–40% (e.g., *C9orf72*+: 37.9% versus *GRN*+: 47.1% in Benussi and colleagues).^17,58,59^ While the NPI-Q affective subsyndrome and the ISPC Distressed subdomain included depression items, they were not significantly different among the groups. A potential reason is that the other items included in the subdomains (e.g., anxiety, easily overwhelmed) may be also present in non-carriers and fluctuate longitudinally.^52^ This suggests that different instruments for the detection of depressive symptoms may have different sensitivity to detect the beginnings of depression during the earliest stages of genetic FTD.

Within a subset of participants with two MRI visits, we investigated whether early changes in structural MRI measures could predict the subsequent changes in NPS scores over the study period. We observed a positive association between the rates of frontal lobar WMSA accumulation between the first two study visits and subsequent increases in NPI-Q total scores over the entire follow-up period. Frontal lobar WMSA are prevalent among FTD patients,^22,60^ especially in *GRN+* where accumulation starts during the presymptomatic stage and progresses throughout the disease course.^44,61^ Studies involving participants with mild cognitive impairment or Alzheimer's disease (AD) have shown positive correlations between WMSA and NPI-Q scores,^22–24^ with evidence of WMSA load being a greater contributor to NPI-Q increases compared to GM atrophy.^24^ These results are in line with our finding that WM alterations could be a potential early marker of NPS. Also, WMSA were not significantly associated with FBI, BDI or ISPC scores in our study, which may be due to 1) the intrinsically small variance of FBI scores among presymptomatic participants, and 2) insignificant WMSA accumulation among *C9orf72+* and non-carriers. While ISPC has been associated with focal vmPFC damage,^34^ such lesions have different implications from conventional WMSA on MRI. Further investigation is warranted to determine whether the relationships among WM abnormalities, tissue atrophy and different NPS measures emerge later in the course of genetic FTD. Moreover, it would be necessary to determine whether WMSA and GM atrophy interact and contribute to additional NPS in the future.

On the other hand, we did not observe significant associations between GM volumes and NPS scores, unlike two previous studies from the Genetic Frontotemporal Dementia Initiative that reported correlations between baseline GM volumes and NPS such as apathy^20^ and psychotic/mood symptoms.^21^ Presymptomatic carriers in the study by Malpetti and colleagues were already developing worsening apathy, suggesting that the participants were further along the disease trajectory. The study by Sellami and colleagues included both symptomatic and presymptomatic carriers; therefore, a direct comparison of previous studies with our study is difficult. GM structural change is among the MRI markers that decline relatively later in the presymptomatic time window,^62,63^ at the global/lobar level, hence an association with NPS changes may not emerge until later in the course of the disease. Our findings suggest that WM pathology could be a more relevant correlate during the earliest stages of NPS development.

Our study had several limitations. First, there was a wide age range among the participants at baseline. While there were no significant group differences in the average baseline age or baseline test scores, it is important to note that this wide age range suggests the participants were likely at various stages of the predementia phase. Additionally, our longitudinal NPS measurements were unbalanced due to varying numbers of follow-up visits among participants. These limitations were partially alleviated by the inclusion of baseline age and scores in the GLME models, which adjust the intercepts according to the baseline variables and can accommodate different numbers of repeated measures.^64^ Second, our MRI measures were based on two time points, which corresponded to the first and second NPS visits. This means we were only able to capture structural changes during the initial phase of the follow-up, and further longitudinal investigation is necessary to reveal anatomical changes that may progress concurrently with NPS changes. Third, our sample size was relatively small due to being a single site study. Although our study design is exempt from potential site-related confounding factors, the analysis likely had reduced power to detect differences among genetic groups. While we only analyzed participants who were deemed ‘predementia’, a future study of individuals who convert from predementia to dementia stages will allow us to observe the NPS and MRI changes during conversion and compare whether they differ among the genetic groups. Also, it would be informative to determine whether specific subsyndromes/subdimensions are better correlated with MRI markers. However, such analyses may be feasible using a larger multi-site sample. Fourth, our *GRN* + were unbalanced in terms of sex and our findings may be weighted towards female characteristics. Additionally, approximately 80% of our *GRN* + were from a single large family, resulting in a limited generalizability of the results. Future analyses are warranted using a multi-site dataset that includes a larger number of independent families to enhance the robustness of the findings. Fifth, while the pathogenic *C9orf72* mutation is characterized by a repeat expansion at a single location, pathogenic *GRN* mutations are far more diverse in their types. For example, in this study, the three families that included *GRN* + participants were each associated with different GRN mutations. This poses an important limitation as the potential effect of the *GRN* mutation types on neuropsychiatric phenotypes was likely confounded by family membership. Also, due to the small sample size of *GRN* + participants, *GRN* mutation types were combined. However, a larger study is warranted to determine if different *GRN* mutation types have distinct associations with NPS progression and brain imaging changes. Sixth, although we acknowledge that we did not exclude the presence of mutations in other dementia-associated genes in our cohort by performing whole-genome or whole-exome sequencing (WGS/WES), the rarity of such mutations in the general population makes this highly unlikely.

### Conclusions

In summary, our findings indicate that the progression of NPS may begin during the predementia stages of FTD, with carriers of FTD mutations exhibiting characteristic changes according to different rating scales. Both *GRN+* and *C9orf72+* showed progression of depression ratings obtained with the BDI. Additionally, *GRN* *+* showed progression of overall personality disturbance ratings, particularly the emotional/social personality subdimension of the ISPC. *C9orf72+* were characterized by progression of ISPC-rated dysexecutive personality disturbances. We also found an overall association between NPI-Q changes and frontal lobar WMSA volume increases, which suggests an anatomical basis for NPS development and progression in FTD mutation carriers. These findings will help characterize the early changes observed in *GRN* and *C9orf72* mutation carriers, which in turn may help to refine the individualized treatment plans for these patients and inform clinical trial design for potential disease modifying therapies.
